# Streptococcus equi subspecies zooepidemicus Meningitis, Septicemia, and Brain Infarcts in a Costa Rican Infant

**DOI:** 10.7759/cureus.17286

**Published:** 2021-08-18

**Authors:** Fabricio Sevilla-Acosta, Angela Ballestero-Pernudi, Elisandro Jiménez-Cruz, Hazel Álvarez-Cabalceta, Gabriela Naranjo-Zuñiga

**Affiliations:** 1 Pediatrics, Hospital Nacional De Niños "Dr. Carlos Sáenz Herrera", San José, CRI; 2 Pediatrics, Hospital La Anexión, Nicoya, CRI; 3 Microbiology, Hospital La Anexión, Nicoya, CRI; 4 Infectious Disease, Hospital Nacional De Niños "Dr. Carlos Sáenz Herrera", San José, CRI

**Keywords:** streptococcus equi, septic shock in children, gram-positive meningitis, infants, brain infarcts, subdural empyema

## Abstract

*Streptococcus equi*, an equine commensal bacterium, is a rare etiology of septicemia and meningoencephalitis in humans and is extremely infrequent in children. Scarce literature has been published about its clinical presentation, treatment and outcomes in infants. Here, we describe a case of *S. equi subsp. zooepidemicus* septicemia and meningoencephalitis in a five-month-old Costa Rican infant that was confirmed by peripheral blood and cerebrospinal fluid (CSF) cultures in a regional hospital of the Pacific coast of Costa Rica who developed multiple ischemic cerebral infarcts secondary to infectious vasculitis, a subdural empyema and serious neurological sequelae. We also reviewed the literature on *S. equi *meningitis in infants under one year of age. This is the first reported case in our country, the fourth reported in infants under one year of age and the second describing multiple cerebral infarcts and subdural empyema in an infant.

## Introduction

*Streptococcus equi* is a beta-hemolytic group C Streptococcus. There are four *Streptococcus* in the Lancefield group C: *S. equi subsp. equi, S. equi subsp. zooepidemicus, S. dysgalactiae subsp. dysgalactiae,* and *S. dysgalactiae subsp. equisimilis* [1]. All of them can cause disease in humans but are considered rare diseases. *S. equi subsp. zooepidemicus* is a zoonotic and opportunistic pathogen closely related to *S. equi subsp. equi*; both share virulence factors with *Streptococcus pyogenes* and can cause severe disease in humans [2].

*S. equi subsp. zooepidemicus *often colonizes and causes disease in horses, but has also been described to cause disease in other mammals (swine, cattle, guinea pigs and sheep). In humans it is an uncommon zoonotic disease with very few reported cases. Infections reported in humans range from mild diseases as pharyngitis, skin and soft tissue infections to severe diseases as epiglottitis, pneumonitis, septic arthritis, osteomyelitis, peritonitis and even bacteremia; endocarditis and meningitis have also been described [1-3]. The source of the infection is associated with direct horse contact, ingestion of unpasteurized dairy products or contact with infected mammals' secretions [1-3]. Most of the infections previously reported occurred in adult patients. *S. equi* infections in children are very rare and it is presumed that children who are immunologically compromised would be more susceptible to infection with this pathogen [1].

World literature describes approximately 50 cases of group C streptococci meningitis, most of them in adult patients [1]. *S. equi subsp. zooepidemicus *appears to be the most common subspecies of group C Streptococcus to cause meningitis [4]. In the literature, we found only three isolated published cases of children under one year of age with *S. equi subsp. zooepidemicus* meningitis [3,5,6]. It is considered a rare disease in all age groups and is associated with poor outcomes [7]. To our knowledge, no other author has analyzed the burden of this disease in children, especially in infants under one year of age.

We describe the case of a healthy Costa Rican infant with septicemia and meningoencephalitis caused by *S. equi subsp. zooepidemicus *confirmed by both peripheral blood and cerebrospinal fluid (CSF) cultures, who developed multiple ischemic cerebral infarcts secondary to infectious vasculitis, a subdural empyema and serious neurological sequelae. This is the first reported case in our country, the fourth reported in infancy in the literature and the second requiring neurosurgical drainage of a brain subdural empyema and describing multiple cerebral infarcts in an infant.

## Case presentation

A five-month-old male was admitted to La Anexión Hospital, a community hospital on the Pacific coast of Costa Rica, with a history of febrile status epilepticus. He had experienced four days of fever and vomiting. On admission, physical examination revealed a febrile encephalopathic infant with obvious signs of intracranial hypertension (altered mental status, setting-sun sign, bulging fontanel, arterial hypertension and bradycardia) associated with focal seizures and septic shock.

He needed immediate endotracheal intubation, mechanical ventilation, intravenous (IV) fluids and antiepileptic drugs (diazepam and phenytoin). Peripheral blood cultures and blood samples were obtained for laboratory analysis. Meningeal doses of intravenous cefotaxime were started as empiric therapy and lumbar puncture was deferred at that moment because of intracranial hypertension.

Complete blood count revealed discrete anemia and leukocytosis with normal platelets (hemoglobin: 10.1 g/dL; leukocytes: 14,490/mm3; platelets: 198,000/mm3). Coagulation profile was slightly abnormal (prothrombin time [PT]: 51%, activated partial thromboplastin time [APTT]: 20s, international normalised ratio [INR]: 1.46). C-reactive protein (CRP) was negative (6 mg/L) and procalcitonin (PCT) was positive (21 ng/mL). Creatinine, blood urea nitrogen, electrolytes and liver enzymes were normal. Respiratory panel was negative including severe acute respiratory syndrome coronavirus 2 (SARS-CoV-2) and brain ultrasound revealed hydrocephalus, without echogenic interior material and no extra-axial collections.

**Figure 1:**
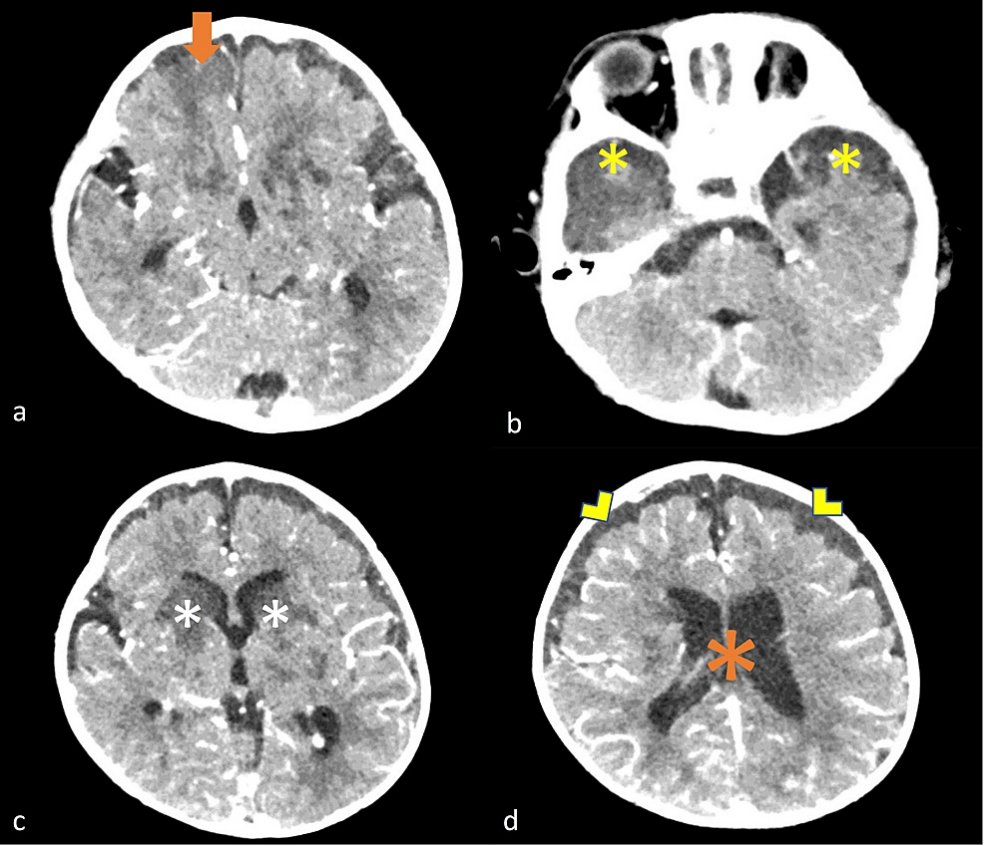
Brain CT scan at admission to the pediatric intensive care unit. a) Hypodense lesions in the right frontal lobe (orange arrow); b) Bilateral temporal lobe hypodense lesions (yellow asterisk); c) Bilateral basal ganglia hypodense lesions (white asterisks); d) Ventriculomegaly (orange asterisk) and early subdural empyema (yellow arrowheads).

Patient was transferred to the pediatric intensive care unit (PICU) of the Hospital Nacional de Niños “Dr. Carlos Sáenz Herrera”, the only national tertiary pediatric center in our country. Brain CT scan showed multifocal hypodense lesions localized in basal ganglia and cerebral cortex (Figure 1). Brain MRI showed T1 hypointense and T2-FLAIR hyperintense lesions localized in bilateral temporal lobes, right frontal lobe, left occipital lobe and basal ganglia (thalamus, caudate nucleus, putamen and globus pallidus). These lesions were apparent diffusion coefficient (ADC) hypointense and diffusion-weighted imaging (DWI) hyperintense, confirming ischemic events secondary to infectious vasculitis.

At the PICU, CSF analysis was obtained and revealed hipoglucorraquia (undetectable glucose), elevated proteins (256 mg/dL), pleocytosis (leucocytes: 265/mm3; 50% lymphocytes; 50% neutrophils) and Gram-positive bacteria in chains on Gram stain. Biofire FilmArray (bioMérieux, Marcy L`Etoile, France) Meningitis/Encephalitis panel was negative for most common pathogens.

**Figure 2:**
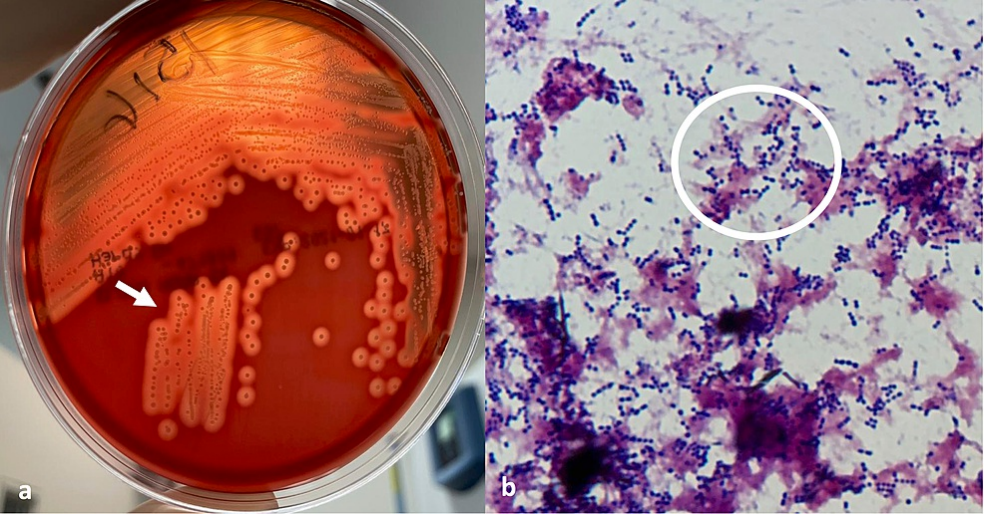
Cultures of peripheral blood. a) Beta-hemolytic large colony of Lancefield group C streptococcus in blood agar (white arrow); b) Growing of multiple Gram-positive streptococci in Gram stain (white circle).

On the second PICU day, blood cultures taken at the referral hospital and CSF taken at the PICU grew large colonies of Lancefield group C beta-hemolytic *Streptococcus*. Further characterization identified the organism as *Streptococcus equi subsp. zooepidemicus*, both in blood smears and CSF (Figure 2). Blood cultures were taken on admission from two different sites and inoculated in a blood agar plate at a temperature of 37°C. Multiple colonies of Gram-positive cocci in chains were seen at the microscope. At 24h of inoculation, there was growing of multiple colonies of streptococci, of transparent aspect with a 1cm diameter of beta-hemolysis. We identified the germ using VITEK 2 (bioMérieux), which detected *Streptococcus equi subsp. zooepidemicus* with a probability of 93% in both blood cultures. Sensitivity testing was done using AST-ST03 card and determined that the organism was sensitive to beta-lactams (minimum inhibitory concentrations [MIC]: ampicillin=0.25µg/ml; penicillin=0.06µg/ml; cefotaxime=0.12µg/ml; ceftriaxone=0.12µg/ml), vancomycin (MIC: 0.25µg/ml), chloramphenicol (MIC: 4µg/ml), linezolid (MIC: 2µg/ml), rifampicin (MIC: 0.06), intermediate to quinolones (MIC: levofloxacin=2µg/ml; moxifloxacin=0.5µg/ml) and resistant only to trimethoprim/sulfamethoxazole (MIC: 160µg/ml). Finally, the result of the blood cultures was confirmed when the same agent was isolated from the CSF at 24h of inoculation.

On the seventh day of PICU stay, patient had neurological deterioration and a new brain CT scan revealed a subdural collection for which a subdural drainage was needed. He completed a total of 10 days of mechanical ventilation, 19 days of cefotaxime, 14 days of vancomycin and seven days of amikacin.

Neurological examination prior to discharge was consistent with serious neurological compromise: axial hypotonus and spastic quadriplegia. Electroencephalogram (EEG) was abnormal with poor organization and slow basal activity but without paroxysms. Hearing screen revealed severe bilateral sensorineural hearing loss. Patient was discharged feeding across nasogastric tube and an intensive physical therapy program was started.

Epidemiological investigation didn’t determine any contact with horses or consumption of unpasteurized milk, but depriving socio-cultural status and unhygienic conditions were seen in their home visit. There was a similar case of a 30-year-old adult in the same geographic area 14 days prior to our patient admission who presented encephalopathic to La Anexión Hospital, and *Streptococcus equi subsp. zooepidemicus* was isolated from CSF as well. He had negative blood cultures and fully recovered after 10 days of IV vancomycin. He required three days of mechanical ventilation and brain CT scan was normal. A common origin couldn’t be determined between these two cases.

Currently, the patient is one year old and has spastic quadriplegia, severe bilateral hearing loss and feeds by gastrostomy tube. Antiepileptic drugs are still needed (carbamazepine) because he had another status epilepticus after discharge and receives intensive physical therapy weekly.

## Discussion

This is the first case of an infant affected by *Streptococcus equi subsp. zooepidemicus* reported in our country. Bloodstream infections and meningoencephalitis in children in Costa Rica are mainly due to *Staphylococcus aureus* and *Streptococcus pneumoniae*. In this particular age group of our patient, *Streptococcus agalactiae* is the leading cause. Especially for this patient, *Streptococcus agalactiae* was highly suspected at the beginning. Blood cultures documented multiple colonies of Gram-positive streptococci with beta-hemolysis, so empiric antibiotics were targeted to the most frequent streptococci in our environment at meningeal doses. Of note, the result of the Biofire FilmArray (bioMérieux) Meningitis/Encephalitis panel made in the CSF was negative, suggesting that the causative agent was not a common *Streptococcus*. Finally, the results of the blood cultures identified *Streptococcus equi subsp. zooepidemicus *and were confirmed when the same agent was isolated from the CSF.

As reported in other cases in humans, *Streptococcus equi* infections have mostly been reported with the subspecies *zooepidemicus*. Clinical scenarios have been: unknown focus bacteremia, meningitis, endocarditis, and there are also cases of aortitis. These case reports found that consumption of unpasteurized milk products and contact with horses or secretions infected with this organism can be determined in the majority of cases and that the fatality rate of these infections has been especially high in humans [8]. In our patient, there was no identifiable contact with animals or with unpasteurized milk products; apparently this baby received only breastfeeding. However, depriving socio-cultural status and unhygienic conditions were seen in their home visit. Costa Rica is a tropical country and especially the Pacific coast of Guanacaste is an area with a lot of livestock activity, so it is possible that somewhere in the patient’s house he could have contact with secretions of this kind of animals or domestic animals, as it is also known that even dogs can transmit the illness [3]. Of especial interest, there was a report of an adult patient living in the same geographic area who developed *Streptococcus equi subsp. zooepidemicus* meningitis in the previous 14 days, suggesting a common origin of both cases, however, this association couldn’t be determined by the epidemiological investigation. It seems that this infection is more severe in children than in adults, as the adult patient fully recovered and our patient had severe neurological sequelae. Probably, the infant developed this catastrophic course of the diseases because of his immature immunological system and a high burden of the pathogen, as demonstrated by the isolation of the pathogen both in blood and CSF.

Other authors have previously performed a literature search and review of *Streptococcus equi* meningitis. van Samkar et al. in 2016 [7] and Torpiano et al. in 2020 [9] made a literature search in PubMed using the terms *“Streptococcus equi" *and *"meningitis”.* We actualized this data with the cases published in PubMed since then and our two cases, with especial interest in the reported cases of infants under one year of age. Using the same words in a literature search in PubMed, actually there are 37 reported cases of *S. equi *meningitis caused by both subspecies between 1978 and 2021, 32 of which were caused by subspecies *zooepidemicus*, and three caused by subspecies *equi* (two cases were reported as *S. equi* meningitis but did not distinguish between subspecies) [1-18]. *S. equi* meningitis in children is especially infrequent as the majority of cases reported have been in adult patients (>70 years: 14 patients; 40-69 years: 10 patients; 20-39 years: six patients; 10-19 years: three patients; 1-9 years: 0 patients), and only four cases were under one year of age [3,5,6,18]. Here we report two more cases: a five-month-old infant and a thirty-year-old male. Combining these data with our two cases, there are now 39 reported *S. equi* meningitis cases, in which seven cases occurred in the pediatric age and five were under one year of age.

There are only six previous pediatric meningitis cases by *S. equi* reported in the literature (see Table 1), including a one-day-old male who died [5], a one-day-old male who recovered completely [18], a 14-week-old male who also recovered completely [18], a six-month-old female who had severe hearing loss and mild neurocognitive deficits [3], a 13-year-old female who had unilateral hearing defect [10] and a 13-year-old male who recovered well without sequelae [9].

**Table 1:** Streptococcus equi meningitis in pediatric patients (sorted by age). CR: Costa Rica; UK: United Kingdom; USA: United States of America. *Current reported case.

#	Country, year	Age	Sex	Subspecies	Disease features	Treatment (Duration)	Outcome	Reference
1	UK, 1984	1 day	Male	Zooepidemicus	Septicemia, Meningitis	Not specified	Died	[5]
2	USA, 1997	1 day	Male	Not specified	Meningitis	Ampicillin (10 days)	Recovered completely	[18]
3	UK, 2000	14 weeks	Male	Zooepidemicus	Septicemia, Meningitis	Ceftriaxone (3 weeks)	Recovered completely	[18]
4	CR, 2021	5 months	Male	Zooepidemicus	Septicemia, Meningitis, Multifocal brain infarctions, Subdural empyema	Cefotaxime (19 days), Vancomycin (14 days), Amikacin (7 days)	Cerebral palsy, severe bilateral hearing loss, epilepsy	[*]
5	USA, 2019	6 months	Female	Zooepidemicus	Septicemia, Meningitis, Multifocal brain infarctions, Subdural empyema	Ceftriaxone (2 days), Vancomycin (2 days), Dexamethasone (2 days), Penicillin G (4 weeks)	Severe hearing loss and mild neurocognitive deficits	[3]
6	USA, 2001	13 years	Female	Zooepidemicus	Septicemia, Meningitis	Ceftriaxone + Vancomycin (14 days)	Unilateral hearing defect	[10]
7	Malta, 2020	13 years	Male	Equi	Septicemia, Meningitis, Subdural empyema	Ceftriaxone + Rifampicin (8 weeks)	Recovered completely	[9]

Our patient is the fifth case of *S. equi *meningitis reported in an infant under one year of age, all of them caused by the subspecies *zooepidemicus,* except one case in which authors did not distinguish between subspecies (Table 1). One patient died (20%), of the surviving four patients, two had neurological sequelae (50%) and two recovered completely (50%). The most common sequela was hearing loss and, unfortunately, our case is the first to report cerebral palsy and epilepsy as permanent neurological sequelae. In this age group, the mortality rate seems to be the same as previously reported in other age groups (20%) but permanent neurological morbidity seems to be higher than reported in adults (50% in infants under one year versus 31.4% in adults) [9].

In previous reviews of *S. equi* meningitis, cranial CT scan showed abnormalities in 50% of patients (brain edema, sinusitis, mastoiditis, brain abscesses, hydrocephalus and cerebral hypodensities were the more common abnormalities), and only two out of 33 patients older than one year developed a subdural empyema (6%), in contrast with infants under one year of age, in which two out of five required neurosurgical drainage of subdural empyema (40%) [7,9]. As bacteremia is more frequent in infants under one year, this could explain the higher incidence of intracranial and subdural complications in this age group. It is known in mouse models of infection that some virulence factors of *S. equi subsp. zooepidemicus* induce meningitis by breaking through the blood-brain barrier (BBB). High bacteremia is necessary for binding to the brain microvascular endothelium of the BBB and inducing meningitis [19]. In our patient multiple ischemic cerebral infarcts secondary to infectious vasculitis were also documented, an intracranial complication reported only in one previous patient. In this age group the brain insult is determinant because an injury to the developing brain can lead to cerebral palsy. As an example, Torpiano et al. reported a case of a 13-year-old male who was complicated by a subdural empyema secondary to *S. equi subsp. equi* meningitis and fully recovered after neurosurgical drainage and antibiotic treatment [9]. Although this case was due to *S. equi subsp. equi*, and our case was due to *S. equi subsp. zooepidemicus*, both were complicated with subdural empyema and required neurosurgical evacuation, but it seems that in our case the worst outcome could be determined because of the age of the child and the associated intracranial complications.

Empirical antibiotic therapy for pyogenic meningitis includes a third-generation cephalosporin, vancomicin and steroids instituted before the first dose of antibiotics until pneumococcus is ruled out [14]. Once the causative microorganism is identified, treatment should be guided by culture sensitivity report. The recommended antimicrobial therapy for streptococcal meningitis caused by an equine pathogen is penicillin [7]. Bactericidal activity against group C streptococci is achieved when adding gentamicin or rifampin to a β-lactam or vancomycin [14]. There are no published data to recommend routine use of corticosteroids in meningitis caused by *S. equi*. In our case the patient received 19 days of cefotaxime, 14 days of vancomycin and seven days of amikacin as adjunctive therapy because of the complication of the subdural empyema. In the literature the duration of antibiotic therapy ranged between 10 days to eight weeks (Table 1) [3,6,9,10,13,14,18].

## Conclusions

We describe the case of a five-month-old male presenting with meningoencephalitis and septicemia by *S. equi subsp. zooepidemicus* who developed multiple ischemic cerebral infarcts secondary to infectious vasculitis, a subdural empyema and serious permanent neurological sequelae. This case warns of a potentially devastating infection by an invasive equine pathogen that should be promptly treated with penicillin or cephalosporin and in the presence of subdural collections may require surgical drainage. Despite rapid institution of appropriate treatment, this infection could lead to death in 20% of patients and severe neurological sequelae in 50% of infants under one year of age, including cerebral palsy, so prevention is the cornerstone of treatment. Prevention of this disease includes avoiding contact with horses and avoiding consumption of unpasteurized cow's milk. This case report and the review of the literature reminds us that, although infrequent, meningoencephalitis by *S. equi* is a serious threat to human health and could lead to permanent neurological sequelae in children, especially in infants under one year of age.
